# Medication patterns and potentially inappropriate medication in patients with metastatic breast cancer: results of the BRE-BY-MED study

**DOI:** 10.1186/s12885-025-13548-8

**Published:** 2025-01-22

**Authors:** Lilly Sophia Brandstetter, Anna Grau, Peter U. Heuschmann, Max Müller-Reiter, Jessica Salmen, Stefan Störk, Achim Wöckel, Jens-Peter Reese

**Affiliations:** 1 Institute for Clinical Epidemiology and Biometry, Julius-Maximilian University Würzburg, Würzburg, Germany; 2 Institute of Medical Data Science, University Hospital Würzburg, Würzburg, Germany; 3 Department of Gynaecology and Obstetrics, University Hospital Würzburg, Würzburg, Germany; 4 Department of Clinical Research & Epidemiology, Comprehensive Heart Failure Center, University Hospital Würzburg, Würzburg, Germany; 5 Department of Internal Medicine I, University Hospital Würzburg, Würzburg, Germany; 6 Faculty of Health Sciences, Technische Hochschule Mittelhessen University of Applied Sciences, Giessen, Germany

**Keywords:** Metastatic breast cancer, Breast cancer therapy, Polypharmacy, Drug burden, Potentially inappropriate medication, Drug-drug interactions, Medication adherence

## Abstract

**Background:**

The treatment of metastatic breast cancer (mBC) focuses on prolonging patient survival, providing adequate symptom management, and maintaining quality of life (QoL). This includes supportive therapy to prevent or treat potential side effects and handle comorbidities. The combination of mBC therapy, supportive therapy, and treatment for comorbidities increases the risk for polypharmacy, potential drug-drug interactions (pDDI), potentially inappropriate medication (PIM), and potentially missing drugs (pMD). Therefore, the aim of this study was to assess medication patterns of mBC patients in routine care within a cohort study from South Germany.

**Methods:**

Between July 2022 and February 2024 individuals with advanced or mBC, aged ≥ 18 years, living in Bavaria, and who gave written informed consent, were included in the BRE-BY-MED “Breast Cancer Care in Bavaria for Patients with Metastatic Disease” cohort study (DRKS00026601). BRE-BY-MED was carried out at the University Hospital Würzburg with the primary aim of estimating the prevalence of guideline-concordant treatment. For the present analysis cross-sectional data from the baseline assessment was used. Medication was extracted from routine medical records. PIM, pDDI and pMD were assessed using established criteria. Polypharmacy was defined as ≥ 5 concomitantly prescribed drugs.

**Results:**

Ninety-three patients with a median age of 57 years (IQR = 48–64 years), were consecutively enrolled in the BRE-BY-MED study. One patient was male. At baseline, a total of 668 drugs were documented for all patients, including 131 unique substances, of which 44% were mBC therapy, 18% supportive therapy and 38% treatment for comorbidities or supplements. Patients took a median of 6 (IQR = 5–9) concomitant drugs. Polypharmacy (i.e. ≥ 5 concomitant drugs) was observed in 80.6% (*n* = 75) of the patients. PIM were documented in 9.7% (*n* = 9), pDDI in 12.9% (*n* = 12) and pMD in 64.5% (*n* = 60) of the patients.

**Conclusion:**

We observed a high drug burden in mBC patients, largely due to treatment for comorbidities. These drugs might not only be associated with additional risk for side effects, pDDI, or PIM use, yet might also contribute to low medication adherence, higher medication costs and impaired QoL. Considering the burden of mBC and the predicted life expectancy, mBC patients might benefit from closer monitoring of their medication.

**Supplementary Information:**

The online version contains supplementary material available at 10.1186/s12885-025-13548-8.

## Background

Metastatic breast cancer (mBC), defined as breast cancer (BC) that has spread to other parts of the body, occurs in about 30% of all cases [1]. mBC is incurable with a median survival time for patients between two and four years [1, 2]. The focus of treatment in mBC is on prolonging patient survival, providing adequate symptom management, and promoting quality of life (QoL) [1, 3, 4]. This also includes supportive therapy to prevent or treat potential side effects [3, 5].

The incidence of mBC increases with increasing age and the majority of mBC patients are ≥ 65 years at diagnosis [6, 7]. Due to their age, mBC patients are at risk of several comorbidities and might often receive treatment for these comorbidities [8, 9]. This might increase the risk for polypharmacy – commonly defined as at least five or more concomitantly prescribed drugs [10] –, potential drug-drug interactions (pDDI), and potentially inappropriate medication (PIM) [9, 11–17]. Polypharmacy, might not only be associated with additional risk of side effects, pDDI, or PIM use, yet might also contribute to low medication adherence, higher medication costs, and impaired QoL [12–20]. Given the predicted life expectancy of mBC patients, the question was raised whether some comorbidity medications could be deprescribed [16, 18].

Two studies reported that more than one third of mBC patients were taking more than ten tablets daily and that 40% of the tablets comprised non-anticancer drugs [16, 18]. About half of these patients suffered from side effects of these treatments [16, 18]. In addition, (m)BC treatments themselves are often characterised by complex dosing regimens and a high potential for drug-drug and drug-food interactions [21–23]. Furthermore, in cancer patients, the disease itself may also influence the pharmacokinetics of drugs [14].

Besides the prescription drugs for mBC treatment, supportive therapy and comorbidities, mBC patients might additionally self-medicate with over-the-counter (OTC) drugs [24]. A special category of OTC drugs is complementary or alternative medicine (CAM) [25]. CAM use is common among cancer patients, as they hope to alleviate therapy-induced toxicity, improve physical health, and increase the chances of the cancer being cured [26–30]. Studies have shown that OTC drugs and CAM use not only contribute to a high drug burden [18], but also result in a higher risk of pDDI, and in reduced adherence to the BC therapy [27, 31].

Evidence on adverse clinical effects of either prescribed comorbidity medications or OTC drugs in cancer patients is scarce and inconsistent [14, 15, 32, 33]. Equal numbers of studies reported significant and insignificant associations, respectively. Furthermore, even fewer studies explored these topics in patients with mBC [16, 18]. Therefore, the aim of this study was to assess medication patterns of mBC patients in routine care within a cohort study from South Germany.

## Methods

### Participants and study design

Data were derived from individuals participating in BRE-BY-MED “Breast Cancer Care in Bavaria for Patients with Metastatic Disease” (DRKS00026601). BRE-BY-MED was a cohort study carried out at the Department of Gynaecology and Obstetrics of the University Hospital Würzburg with the primary aim of estimating the prevalence of guideline-concordant treatment [34]. In the present analysis cross-sectional data from the baseline assessment was included (see data collection).

### Inclusion and exclusion criteria

BRE-BY-MED included patients of both sexes, with prevalent or newly diagnosed advanced or mBC (hereafter referred to as mBC patients), aged ≥ 18 years, living in Bavaria, and who gave written informed consent to participate. Advanced or mBC was defined by ICD-10 Code C50 or C76-C80 and TNM classification pTx-pNx-M1 or pTx-pN(1–3)-Mx. As patients with both, prevalent and newly diagnosed advanced or mBC, were included, the patients could be at any point in the course of therapy. Exclusion criteria were minimized to age (< 18 years), disease (non-advanced or non-mBC), and not living in Bavaria, to guarantee a most representative study population of clinical routine care.

### Ethics approval and consent to participate

The study was performed in full accordance with the principles of the Declaration of Helsinki (as amended in Tokyo, Venice and Hong Kong). All methods of the study were fully approved by the ethics committee of the Medical Faculty of the University Wuerzburg (reference number 137/21). Written informed consent was obtained from all participants. The responsible data protection officer accepted the data management concept.

### Data collection

In July 2022, we conducted a pilot study that proved the feasibility of the study design. Between September 2022 and February 2024, patients diagnosed with mBC were informed by the study personnel about the BRE-BY-MED study and asked to participate. After written informed consent was obtained, patients completed the baseline survey comprising information on sociodemographic factors (i.e. age, sex, living with a partner, education level, type of health insurance, smoking status, BMI, support by relatives), and several patient reported outcomes (PROs) (see below). In addition, patients were asked if they had ever used CAM. The treating physicians entered clinical information (i.e. Charlson Comorbidity Index (CCI) [35], hormonal receptor (HR) and human epidermal growth factor receptor 2 (HER2) status, diagnosis and localisation of metastasis, current recommendation for additional therapies) into an electronic case report form (eCRF). Medications were manually extracted from routine medical records and were entered into the eCRF by study personnel (LB), trained by an experienced physician of the Department of Gynaecology and Obstetrics of the University Hospital Würzburg (MMR). These included letters/notes from treating physicians (inside and outside University Hospital Würzburg) and the chemotherapy outpatient clinic, tumour board reports, and medication plans (if available). The medication information represent medications prescribed, with assumption that patients actually took those medications.

### Assessment of patient reported outcomes

Perceived health status and QoL were assessed using the EORTC-QL-2 (questions 29 and 30 of the EORTC-QLQ-C30, version 3 [36, 37]). Both items were measured on a 7-point Likert scale (1 = very poor to 7 = excellent). Depressive and anxiety symptoms were assessed using the PHQ-4 [38]. The PHQ-4 consists of the PHQ-2, which measures depressive symptoms, and the GAD-2, which measures anxiety symptoms. For the present analysis for both scales, summary scores were dichotomised using the established cut-off score of ≥ 3 points [38]. Physical functioning was assessed using the PROMIS® v2.0 Physical Function SF 4a (PROMIS-PF-4a) and reported as T-score (range 0 to 100; standardized to the U.S. general population) [39, 40]. The higher the T-score, the worse the physical function [39, 40]. Currently experienced pain level was measured on an 11-point Likert scale (0 = no pain to 10 = strongest pain).

Assessed side effects included infections, metabolic and nutritional disorders, cardiovascular side effects, side effects of the kidney or urinary tract, side effects of the respiratory system, side effects of the eyes, side effects of the nervous system, side effects of the gastrointestinal system, side effects of the skin, and side effects of the musculo-skeletal system. Side effects were chosen based on expert opinion. The impairment caused by currently experienced side effects was measured on a 5-point Likert scale (1 = no impairment to 5 = severe impairment) (see additional file 1). For the present analysis, the impairment was dichotomised by at least slight impairment (= 3 points).

### Classification of drug types

Drugs were classified using the ATC code [41] and grouped by level 2 of the code. In addition, drug types were categorised and grouped by medical function or metabolic system (i.e. mBC therapy, symptomatic/supportive therapy, vitamins/minerals/supplements, cardiovascular system, gastrointestinal system, endocrine system, respiratory system, central nervous system (CNS), psychiatric medicine).

### Identification of potentially inappropriate medication, potential drug-drug interactions, and potentially inappropriate medication

For PIM, the associated risks of adverse events outweigh the potential clinical benefits, particularly when there is evidence in favour of a safer or more effective alternative therapy for the same condition [42, 43]. These drugs should especially not be prescribed in the older population (≥ 65 years) [43]. Criteria to assess PIM and to improve patients’ pharmacotherapy might serve as a helpful guideline [44]. Explicit criteria are mostly drug- or disease-oriented and can be applied with little or no clinical judgement of the patients’ medication history [45]. Implicit criteria are judgment based and therefore, additional clinical information is necessary [45]. We identified explicit PIM according to the PRISCUS list [46].

Explicit pDDI were identified according to the STOPP/START Criteria (version 3) [47], and the Beers Criteria [48] (see additional file 2). Explicit pMD were identified according to the STOPP/START Criteria (version 3) [47], the S3 Guideline for Diagnostic, Treatment and Follow-up Care of Breast Cancer [3], and the S3 Guideline for Supportive Therapy for Oncological Patients [5] (see additional file 3).

### Statistical analyses

Descriptive statistics were computed for the characteristics of the study population, the number of drugs per patient, the observed drug types, PIM, pDDI, and pMD. Continuous data were reported as median (25–75% Inter Quartile Range (IQR)), and categorical data as count (percentage).

### Subgroup analyses

To identify potential influencing factors from PROs, sociodemographics, and clinical characteristics (see data collection) for polypharmacy, PIM, pDDI, and pMD, descriptive subgroup analyses were performed. For these analyses the CCI was classified into two groups (mild = CCI score 1–2, moderate-severe = CCI score ≥ 3) [35]. The BMI was classified into 4 groups (< 18.5 points = underweight, 18.5–24.9 points = normal weight, 25.0–29.9 points = overweight, ≥ 30.0 points = obese). The time since first diagnosis of metastasis, number of metastases occurred during the diseases course, number of concurrent metastases, perceived health status, QoL, physical functioning, and pain level were each classified into two groups using the median split.

## Results

### Characteristics of study participants

**Fig. 1:**
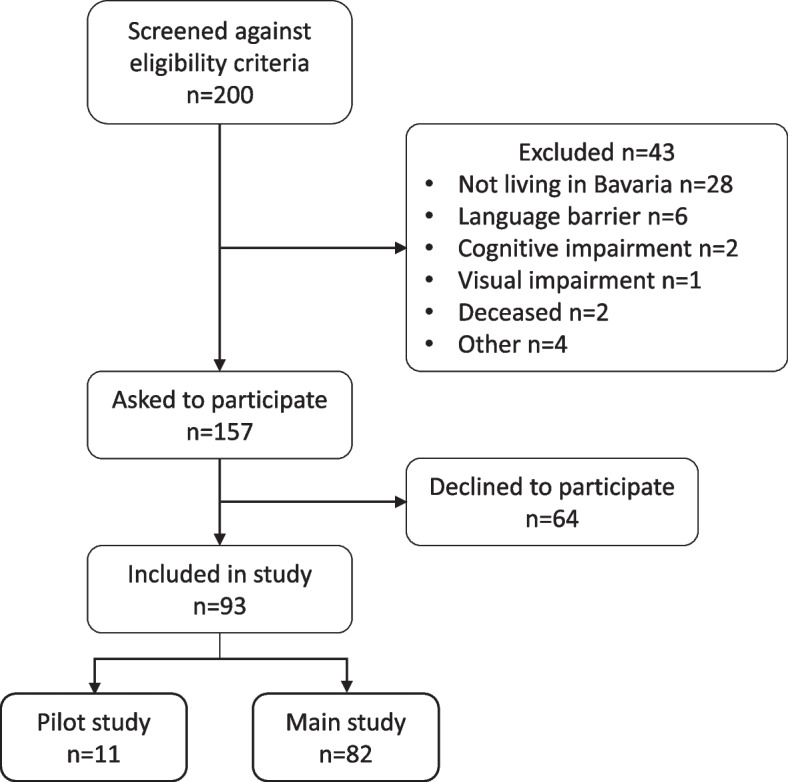
Flow-chart of the BRE-BY-MED study

Ninety-three patients were consecutively enrolled in the BRE-BY-MED study, of whom 11 were included in the pilot study and 82 in the main study. The study flow-chart is presented in Fig. 1.

The median age was 57 years (IQR = 48–64 years), and one patient was male (Table 1). 23.7% of the patients were aged ≥ 65 years. The median perceived health status and the QoL were moderate, with 4.0 (IQR = 3.8–5.0) and 4.0 (IQR = 4.0–5.0) points, respectively. In 25.8% and 17.2% of the patients, respectively, there was evidence for severe depressive or anxiety symptoms according to the cut-off of ≥ 3 points for both scales. At least slight impairment due to side effects of the musculo-skeletal system (57.0%), and side effects of the skin/hair loss (55.9%) were most frequently reported by the patients, followed by impairment due to side effects of the gastrointestinal system (*n* = 45, 48.4%), cardiovascular side effects (44.1%), and side effects of the nervous system (40.8%).

**Table 1:** Characteristics of the study population and subgroup analyses (*n* = 93)

	**Number of patients (n, %)**	**Number of drugs (median, IQR)**	**Polypharmacy**^**a**^ **(n, %)**	**PIM**^**a**^ **(n, %)**	**Potential DDI**^**a**^ **(n, %)**	**Potentially missing drugs**^**a**^ **(n, %)**
***All Patients***	93 (100)	6.0 (5.0–9.0)	75 (80.6)	9 (9.7)	6 (6.5)	49 (52.7)
***Age groups***						
18–49 years	28 (30.1)	6.0 (5.0–8.0)	22 (78.6)	4 (14.3)	1 (3.6)	15 (53.6)
50–64 years	43 (46.2)	6.0 (5.0–9.5)	33 (76.7)	2 (4.7)	4 (9.3)	27 (62.8)
≥ 65 years	22 (23.7)	7.0 (5.3–9.0)	20 (90.9)	3 (13.6)	1 (4.5)	7 (31.8)
***Living in a partnership**** (missing n* = *18)*						
Yes	56 (60.2)	6.0 (5.0–9.0)	44 (78.6)	5 (8.9)	4 (7.1)	30 (53.6)
No	19 (20.4)	7.0 (5.0–9.0)	17 (89.5)	1 (5.3)	2 (10.5)	11 (57.9)
***Supported by relative**** (missing n* = *11)*						
Yes	72 (77.4)	6.0 (5.0–9.0)	56 (77.8)	7 (9.7)	5 (6.9)	36 (50.0)
No	10 (10.8)	6.0 (5.0–7.0)	9 (90.0)	0 (0)	0 (0)	8 (80.0)
***Education level**** (missing n* = *1)*						
Grammar school	28 (30.1)	6.5 (5.0–9.0)	22 (78.6)	2 (7.1)	0 (0)	17 (60.7)
Secondary school	72 (77.4)	6.0 (5.0–9.0)	52 (72.2)	6 (9.4)	6 (9.4)	31 (48.4)
***Health care insurance**** (missing n* = *2)*						
Statutory	82 (88.2)	6.0 (5.0–9.0)	65 (79.3)	7 (8.5)	6 (7.3)	41 (50.0)
Private	9 (9.7)	9.0 (5.5–11.0)	8 (88.9)	1 (11.1)	0 (0)	7 (77.8)
***Smoking status**** (missing n* = *3)*						
Smoker (active/ former/passive)	50 (53.8)	6.5 (5.0–10.0)	42 (84.0)	4 (8.0)	4 (8.0)	29 (58.0)
Never smoked	40 (43.0)	6.0 (5.0–8.0)	31 (77.5)	4 (10.0)	2 (5.0)	17 (42.5)
***BMI**** (missing n* = *8)*						
Underweight	4 (4.3)	4.5 (2.3–9.8)	2 (50.0)	0 (0)	0 (0)	2 (50.0)
Normal weight	32 (34.4)	6.0 (5.0–8.8)	25 (78.1)	2 (6.3)	2 (6.3)	14 (43.8)
Overweight	30 (32.3)	7.0 (5.0–9.0)	25 (83.3)	3 (10.0)	3 (10.0)	17 (56.7)
Obese	19 (20.4)	5.0 (5.0–9.0)	16 (84.2)	2 (10.5)	1 (5.3)	9 (47.4)
***Charlson Comorbidity Index (CCI)***						
CCI 1–2 points	78 (83.9)	6.0 (5.0–9.0)	61 (78.2)	5 (6.4)	6 (7.7)	42 (53.8)
CCI ≥ 3 points	15 (16.1)	8.0 (5.5–9.0)	14 (93.3)	4 (26.7)	0 (0)	7 (46.7)
***Menopausal status***						
premenopausal	27 (29.0)	6.0 (5.0–8.0)	21 (77.8)	2 (7.4)	2 (7.4)	16 (59.3)
perimenopausal	2 (2.2)	4,5 (4.0–4.5)	1 (50.0)	0 (0)	0 (0)	1 (50.0)
postmenopausal	60 (64.5)	6.0 (5.0–9.8)	49 (81.7)	5 (8.3)	4 (6.7)	30 (50.0)
unknown	3 (3.2)	5.0 (5.0–5.0)	3 (100)	2 (66.7)	0 (0)	2 (66.7)
Male patient	1 (1.1)	*(8.0)*	*1 (100)*	*1 (100)*	*0 (0)*	*0 (0)*
***HR status**** (missing n* = *1)*						
HR +	72 (78.3)	6,5 (5.0–9.0)	57 (79.2)	8 (11.1)	2 (2.8)	37 (51.4)
HR-	20 (21.7)	6.0 (5.0–10.3)	17 (85.0)	1 (5.0)	4 (20.0)	12 (60.0)
***HER2 status**** (missing n* = *2)*						
HER2 +	21 (23.1)	5.0 (5.0–7.0)	16 (76.2)	3 (14.3)	1 (4.8)	13 (61.9)
HER2-	70 (76.9)	7.0 (5.0–9.0)	57 (81.4)	6 (8.6)	5 (7.1)	35 (50.0)
***Time since first diagnosis of metastasis***						
0 years	63 (67.7)	7.0 (5.0–9.0)	52 (82.5)	6 (9.5)	4 (6.3)	26 (51.0)
≥ 1 year	30 (32.3)	5.0 (4.0–8.3)	23 (76.7)	3 (10.0)	2 (6.7)	23 (54.8)
***Number of metastases occurred during the course of the disease***						
≤ 2 metastases	63 (67.7)	6.0 (5.0–9.0)	52 (82.5)	6 (9.5)	4 (6.3)	35 (55.6)
≥ 3 metastases	30 (32.3)	7.0 (4.8–10.3)	23 (76.7)	3 (10.0)	2 (6.7)	14 (46.7)
***Number of concurrent metastases***						
1 metastasis	55 (59.1)	6.0 (5.0–9.0)	44 (80.0)	5 (9.1)	1 (1.8)	33 (60.0)
≥ 2 metastases	38 (40.9)	6.0 (5.0–9.3)	31 (81.6)	4 (10.5)	5 (13.2)	16 (42.1)
***Localisation of metastases***^**b**^						
axillary	34 (36.6)	7.0 (5.0–9.0)	30 (88.2)	0 (0)	1 (2.9)	21 (61.8)
hepatic	24 (25.8)	7.0 (5.0–11.0)	19 (79.2)	1 (4.2)	4 (16.7)	15 (62.5)
pulmonary	17 (18.3)	5.0 (5.0–9.0)	10 (58.8)	3 (17.6)	1 (5.9)	9 (52.9)
pleural	9 (9.7)	5.0 (4.5–8.5)	7 (77.8)	2 (22.2)	0 (0)	4 (44.4)
peritoneal	1 (1.1)	*(4.0)*	*0 (0)*	*0 (0)*	*0 (0)*	*0 (0)*
lymphogenic	21 (22.6)	7.0 (5.0–10.5)	18 (85.7)	2 (9.5)	2 (9.5)	10 (47.6)
cerebral	8 (8.6)	6.0 (5.0–7.8)	7 (87.5)	3 (37.5)	1 (12.5)	3 (37.5)
ossary	34 (36.6)	6.0 (5.0–9.0)	28 (82.4)	5 (14.7)	3 (8.8)	13 (38.2)
***Additionally recommended therapy***^**c**^						
None	15 (16.1)	5.0 (4.0–6.0)	9 (60.0)	1 (6.7)	0 (0)	11 (73.3)
Mastectomy	8 (8.6)	9.0 (7.3–9.8)	8 (100.0)	0 (0)	0 (0)	8 (100.0)
Lymphadenectomy/ axillary debulking	17 (18.3)	8.0 (6.5–10.0)	16 (94.1)	0 (0)	0 (0)	14 (82.4)
Surgical removal of metastases	10 (10.8)	4.0 (3.0–6.3)	4 (40.0)	1 (10.0)	0 (0)	5 (50.0)
Radiotherapy	52 (55.9)	7.0 (5.0–9.0)	46 (88.5)	5 (9.6)	3 (5.8)	28 (53.8)
Psycho-oncology	32 (34.4)	7.0 (5.0–10.5)	29 (90.6)	6 (18.8)	4 (12.5)	15 (46.9)
Palliative care	27 (29.0)	6.0 (5.0–11.0)	23 (85.2)	3 (1.1)	3 (1.1)	12 (44.4)
Physiotherapy	33 (35.5)	8.0 (6.0–10.0)	31 (93.9)	5 (15.2)	2 (6.1)	19 (57.6)
***Perceived health status – EORTC-QL-2**** (missing n* = *3)*						
Health status < 4	22 (23.7)	8.0 (5.0–10.3)	21 (95.5)	4 (18.2)	3 (13.6)	13 (59.1)
Health status ≥ 4	68 (73.1)	6.0 (5.0–8.8)	52 (76.5)	3 (4.4)	3 (4.4)	35 (51.5)
***Quality of life (QoL) – EORTC-QL-2**** (missing n* = *3)*						
QoL < 4	20 (21.5)	8.0 (7.0–11.0)	19 (95.0)	4 (20.0)	3 (15.0)	13 (65.0)
QoL ≥ 4	70 (75.3)	6.0 (5.0–8.3)	54 (77.1)	3 (4.3)	3 (4.3)	35 (50.0)
***Depressive symptoms – PHQ-2**** (missing n* = *1)*						
Summary score < 3	68 (73.1)	6.0 (5.0–9.0)	53 (77.9)	4 (5.9)	3 (4.4)	34 (50.0)
Summary score ≥ 3	24 (25.8)	7.5 (5.0–9.0)	21 (87.5)	4 (16.7)	3 (12.5)	14 (58.3)
***Anxiety symptoms – GAD-7**** (missing n* = *1)*						
Summary score < 3	76 (81.7)	6.0 (5.0–9.0)	61 (80.3)	7 (9.2)	4 (5.3)	40 (52.6)
Summary score ≥ 3	16 (17.2)	7.0 (5.0–8.8)	13 (81.3)	1 (6.3)	2 (12.5)	8 (50.0)
***Physical functioning – PROMIS-PF-4a**** (missing n* = *2)*						
T-score < 47	48 (51.6)	6.0 (4.0–7.8)	35 (72.9)	4 (8.3)	1 (2.1)	22 (45.8)
T-score ≥ 47	43 (46.2)	7.0 (5.0–11.0)	38 (88.4)	4 (9.3)	5 (11.6)	26 (60.5)
***Experienced pain level**** (missing n* = *2)*						
Pain level < 2	35 (37.6)	6.0 (4.0–9.0)	26 (74.3)	1 (2.9)	2 (5.7)	17 (48.6)
Pain level ≥ 2	56 (60.2)	6.5 (5.0–9.0)	47 (83.9)	7 (12.5)	4 (7.1)	30 (53.6)
***At least slight impairment due to side effects***^**d**^						
Infections	23 (24.8) *(missing n* = *11)*	8.0 (4.0–11.0)	17 (73.9)	2 (8.7)	3 (13.0)	15 (65.2)
Metabolic and nutritional disorders	33 (35.4) *(missing n* = *11)*	7.0 (5.0–10.0)	27 (81.2)	6 (18.2)	3 (9.1)	20 (60.6)
Cardiovascular side effects	19 (20.4) *(missing n* = *11)*	7.0 (5.0–10.0)	16 (84.2)	2 (10.5)	1 (5.3)	14 (73.7)
Side effects of the kidney or urinary tract	41 (44.1) *(missing n* = *12)*	7.0 (5.0–10.0)	35 (85.4)	6 (14.6)	3 (7.3)	22 (53.7)
Side effects of the respiratory system	26 (28.0) *(missing n* = *13)*	7.0 (5.0–10.0)	21 (80.8)	3 (11.5)	1 (3.8)	16 (61.5)
Side effects of the eyes	31 (33.4) *(missing n* = *11)*	6.0 (4.0–10.0)	22 (71.0)	3 (9.7)	1 (3.2)	18 (58.1)
Side effects of the nervous system	38 (40.8) *(missing n* = *10)*	7.0 (5.0–10.3)	33 (86.8)	4 (10.5)	4 (10.5)	23 (60.5)
Side effects of the gastrointestinal system	45 (48.4) *(missing n* = *10)*	6.0 (5.0–10.5)	36 (80.0)	3 (6.7)	4 (8.9)	25 (55.6)
Side effects of the skin/hair loss	52 (55.9) *(missing n* = *10)*	7.0 (5.0–9.8)	43 (82.7)	5 (9.6)	5 (9.6)	29 (55.8)
Side effects of the musculo-skeletal system	53 (57.0) *(missing n* = *12)*	7.0 (5.0–10.0)	44 (83.0)	7 (13.2)	5 (9.4)	27 (50.9)

^a^Proportion of patients with polypharmacy/ PIM/ pDDI/ pMD

^b^More than one loci per patient possible

^c^More than one additional therapy per patient possible

^d^More than one side effect per patient possible

### Medication patterns and drug types

A total of 668 drugs were documented for all patients, including 131 unique substances. The median number of drugs per patient (incl. BC therapy and PRN (pro-re-nata)/on-demand medication) was 6 (IQR = 5–9) with a minimum of two and a maximum of 24 concomitant drugs. 44.0% (*n* = 293) were classified being prescribed for mBC therapy and 11.4% (*n* = 76) were PRN medication. Polypharmacy was observed in 80.6% (*n* = 75) of the patients.

**Fig. 2:**
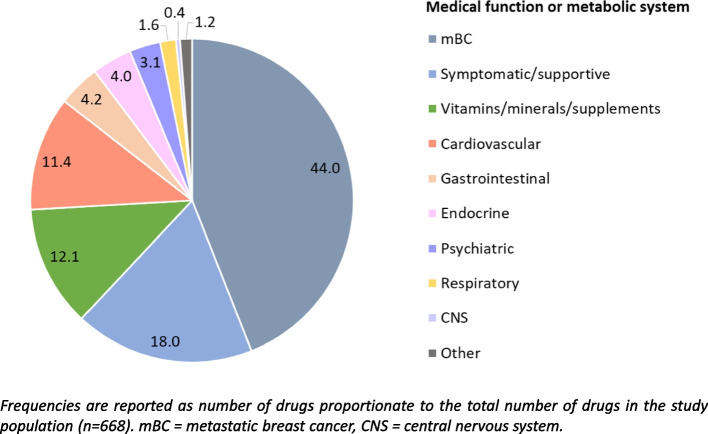
Frequencies of drug types by medical function or metabolic system in the study population (*n* = 93)

According to ATC code level 2, 30 different drug types were observed (see Table 2). mBC therapy was the biggest category followed by symptomatic/supportive therapy, vitamins/minerals/supplements, drug for the cardiovascular system, drugs for the gastrointestinal system, drugs for the endocrine system, and psychiatric medicine (Fig. 2). The smallest categories were drugs for the respiratory system or CNS, and other drugs. A detailed description of the observed drug types is available in the additional file 4.

**Table 2:** Frequencies of drugs (ATC code level 2) and assigned drug types in the study population (*n* = 93)

Drug/drug type	ATC codes (level 2)	Frequency^a^ (n, %)	Drug type by medical function or metabolic system
Antineoplastic agents	L01	127 (19.0)	mBC therapy
Endocrine therapy	L02	113 (17.0)	mBC therapy
Analgesics	N02, M01	69 (10,3)	Symptomatic/supportive therapy
Drugs affecting bone structure and mineralisation	M05	51 (7,6)	mBC^b^ therapy
Minerals	A12	45 (6,7)	Vitamins/minerals/supplements
Antiemetics	A04, H02, D07	44 (6,6)	Symptomatic/supportive therapy
Antihypertensive drugs	C02, C03, C09	30 (4,5)	Drugs for the cardiovascular system
Vitamins	A11	29 (4,3)	Vitamins/minerals/supplements
Thyroid therapy	H03	26 (4)	Drugs for the endocrine system
Proton pump inhibitors (PPI)	A02	25 (3,8)	Drugs for the gastrointestinal system
Antithrombotic agents	B01	18 (2,7)	Drugs for the cardiovascular system
Beta-blocking agents	C07	12 (1,8)	Drugs for the cardiovascular system
Drugs for obstructive airway diseases	R03	11 (1,6)	Drugs for the respiratory system
Psychoanaleptics	N06	11 (1,6)	Psychiatric medicine
Psycholeptics	N05	10 (1,5)	Psychiatric medicine
Lipid modifying agents	C10	7 (1,1)	Drugs for the cardiovascular system
Nutritional supplements	V06	7 (1,1)	Vitamins/minerals/supplements
Antianaemic agents	B03	5 (0,7)	Drugs for the cardiovascular system
Laxatives	A06	5 (0,7)	Symptomatic/supportive therapy
Antiepileptics	N03	3 (0,4)	Drugs for the CNS^c^
Calcium channel blockers	C08	3 (0,4)	Drugs for the cardiovascular system
Gynaecologic agents	G01, G02, G04	3 (0,4)	Other
Homeopathic agents	V60	3 (0,4)	Other
Immunostimulants	L03	3 (0,4)	mBC therapy
Antidiabetics	A10	2 (0,3)	Drugs for the gastrointestinal system
Antidiarrhoeals	A07	2 (0,3)	Symptomatic/supportive therapy
Antigout agents	M04	1 (0,2)	Other
Antimycotics	J02	1 (0,2)	Other
Bile and liver therapy	A05	1 (0,2)	Drugs for the gastrointestinal system
Vasoprotectives	C05	1 (0,2)	Drugs for the cardiovascular system

^a^The frequency is reported as number of drugs proportionate to the total number of drugs in the study population (*n* = 668)

^b^mBC = metastatic breast cancer

^c^CNS = central nervous system

### Potentially inappropriate medication

PIM according to the PRISCUS list were documented in 9.7% (*n* = 9) of the patients, of which one patient took two (Table 3). Five (50%) of the PRISCUS drugs were PRN medications. Three of these patients were aged ≥ 65 years. The PIM in the patients aged ≥ 65 years were exclusively psycholeptics agents (i.e. clobazam, zolpidem, lorazepam), one of which was PRN medication.

**Table 3:** PIM identified in the study population (*n* = 93)

PIM	ATC code	Drug type	ATC code level 2	Frequency^a^ (n, %)
Lorazepam	N05BA06	Psycholeptics	N05	5 (5.4)
Zolpidem	N05CF02	Psycholeptics	N05	2 (2.2)
Clobazam	N05BA09	Psycholeptics	N05	1 (1.1)
Dimenhydrinate	A04AB02	Antiemetics	A04	1 (1.1)
Methyldopa	C02AB	Antihypertensive drugs	C02	1 (1.1)

^a^The frequency is reported as number of patients proportionate to the total number of patients in the study population (*n* = 93)

pDDI according to STOPP-START and Beers Criteria were identified in 12.9% (*n* = 12) of the patients (Table 4). One of the observed pDDI included a PRN medication. In 9.0% (*n* = 2) of the 22 patients aged ≥ 65 years pDDI were observed. These comprised the concomitant use of an opioid and gabapentine and the concomitant use of three CNS-active drugs.

**Table 4:** pDDI identified in the study population (*n* = 93)

Substance 1	ATC code 1	Substance 2	ATC code 2	Frequency^a^ (n, %)
Opioid	N02A	Gabapentin, Pregabaline	N02BF01, N02BF02	5 (5.4)
≥ 3 CNS-active drugs (Antiepileptics, Gabapentinoids, Antidepressants (TCAs, SSRIs, and SNRIs), Antipsychotics, Benzodiazepines, Z-drugs, Opioids, Skeletal muscle relaxants)^b^	N02A, N02BF, N03A, N05A, N05BA, N05CF, N06AA09, N06AB, N06AX16, N06AX21, M03B	-	-	5 (5.4)
Non-steroidal anti-inflammatory drug (NSAID)	M01A	Factor Xa Inhibitor	B01AF	1 (1.0)
Opioid	N02A	Benzodiazepine	N05BA	1 (1.0)

^a^The frequency is reported as number of patients proportionate to the total number of patients in the study population (*n* = 93)

^b^CNS = central nervous system, TCAs = tricyclic antidepressants, SSRIs = selective serotonin reuptake inhibitors, SNRIs = serotonin–norepinephrine reuptake inhibitors

pMD according to STOPP-START Criteria and the S3-Guidelines were identified in 52.7% (*n* = 49) of the patients (Table 5). Regarding the STOPP/START Criteria, in four (36.4%) of the patients the pMD included a PRN medication. According to the STOPP/START Criteria, pMD were observed in 9.0% (*n* = 2) of the 22 patients aged ≥ 65 years. These comprised a missing laxative in two of three (66.7%) patients taking opioids.

**Table 5:** pMD identified in the study population (*n* = 93)

Present drug		Potentially missing drug		Frequency of potential missingness^a^ (n of relevant cases, %)
**Drug**	**ATC code**	**Drug/drug type**	**ATC code**	
non-steroidal anti-inflammatory drug (NSAID)	M01A	Proton-pump inhibitor (PPI)	A02BC	5 of 16 (31.3)
Opioid	N02A	Laxative	A06A	6 of 10 (60.0)
Capecitabine	L01BC06	Antiemetic agent^b^	A04AA01/ H02AB02/ A03FA01/ A04AB02/ N05AH03/ N05AD01/ N05AA02/ N05BA06/ N05BA12	2 of 2 (100)
Pertuzumab	L01FD02			2 of 2 (100)
Lapatinib	L01EH01			1 of 1 (100)
Trastuzumab-Emtansin	L01FD03			1 of 1 (100)
Cyclophosphamid	L01AA01			9 of 17 (53.0)
Epirubicin	L01DB03			9 of 17 (53.0)
Carboplatin	L01XA02			2 of 4 (50.0)
Doxorubicin	L01DB01			2 of 4 (50.0)
Docetaxel	L01CD02			1 of 2 (50.0)
Paclitaxel	L01CD01			12 of 35 (34.3)
Paclitaxel	L01CD01	Antineurotoxic^c^ agent	N06AX16/ N06AA09/ N02BF01/ N02BF02	29 of 35 (82.9)
Docetaxel	L01CD02			1 of 2 (50.0)
Docetaxel	L01CD02	Loperamide	A07DA03	2 of 2 (100)
Bevacizumab	L01FG01			1 of 1 (100)
Lapatinib	L01EH01			1 of 1 (100)
Aromatase inhibitor	L02BG	Vitamin D3/ bisphosphonate/ denosumab	A11CC05/ M05BA/ M05BX04	8 of 35 (22.9)

^a^The frequency is reported as number of patients proportionate to the number of relevant patients (patients with present drug) in the study population (*n* = 93)

^b^Ondansetron/ Dexamethasone/ Metoclopramide/ Dimenhydrinate/ Olanzapine/ Haloperidol/ Levomepromazine/ Lorazepam/ Alprazolam

^c^Venlafaxine/ Amitriptyline/ Gabapentin/ Pregabaline

### Patient reported use of complementary or alternative medicine (CAM)

30.1% (*n* = 28) of the patients reported to have ever used CAM including naturopathic treatments, mistletoe therapy and homeopathic treatments (missing *n* = 2, 2.2%).

### Subgroup analyses

The results of the subgroup analyses are presented in Table 1. In patients with the following characteristics a higher frequency of polypharmacy was observed: older age, living alone, not being supported, private insurance, a BMI indicating overweight, a CCI ≥ 3 points, de novo diagnosis of mBC, axillary, lymphogenic, or cerebral metastases, current recommendations for a mastectomy or lymphadenectomy/ axillary debulking, lower perceived health status, lower QoL and mental health, and worse physical functioning. In patients with lower QoL and mental health, and higher pain level, a higher frequency of PIM and pDDI was observed. In patients with younger age, higher education level, private insurance, axillary or hepatic metastases, and worse physical functioning a higher frequency of pMD was observed.

Regarding the reported side effects, patients who were at least slightly impaired by infections took one to two more drugs than patients who were impaired by other side effects. Polypharmacy was most common in patients at least slightly impaired by side effects of the kidney or urinary tract or side effects of the nervous system. No statistical testing of the found differences was performed.

Concerning smoking status, menopausal status, HR status, HER2 status, the number of metastases that occurred during the course of the disease, and the number of concurrent metastases no trends in number of drugs, PIM, pDDI, or pMD were observed.

## Discussion

### Medication patterns and potentially inappropriate medication

The aim of this study was to assess medication patterns and potentially inappropriate medication in a German cohort of mBC patients using data from routine medical records. We observed a substantial drug burden in mBC patients with a median of six concomitant drugs per patient and polypharmacy was present in 80.6% of the patients (see Table 1). This frequency is partly comparable with the figures reported in systematic reviews of patients with different types of cancer, as the frequencies in the included studies vary widely (2–91%) [32, 49, 50].

The high drug burden might be problematic as more than a quarter of the documented drugs represent treatment for comorbidities, such as drugs for the cardiovascular, endocrine, or gastrointestinal system, or vitamins/minerals/supplements. This may be particularly problematic as medication for comorbidities are rarely considered in clinical trials [51].

However, we identified only 10 PIMs in 9.7% of the patients and 12 pDDI in 12.9% of the patients. The frequency of PIM use was substantially lower than that reported by two systematic reviews of older cancer patients (10.8–57.5%) [49, 50]. Likewise, several studies on pDDI in (older) cancer patients identified higher frequencies (18.3–75.4%) [52–55]. However, these studies did not use DDI criteria, but applied a drug interaction software. The applied software incorporated clinical information, thereby enabling the identification of implicit PIM and DDI, which in turn might explain the higher frequencies. In addition, half of the PIM were PRN medications. Hence, these PIM might be less problematic, as the patients do not take them regularly. The PIM in patients aged ≥ 65 years comprised solely psycholeptic agents (benzodiazepines (BZD) and z-drugs). The pDDI mostly involved CNS-active drugs. These observations are comparable to those of studies including older patients with different cancer types [52–57]. BZD and z-drugs are commonly used to treat insomnia and anxiety in older patients [58, 59]. However, a variety of side effects may occur, including the risk of falls and fractures, cognitive difficulties, drug abuse and dependence [46, 48, 60–62]. Hence, international guidelines recommend short-term use only, and other treatment options should be considered first [63]. Yet, in clinical practice, long-term use seems to increase with age [64, 65]. Due to the cross-sectional nature of our study, we could not determine whether the mBC patients in the present cohort were long- or short-term users.

Given the magnitude of the observed drug burden, questions may be raised about the relevance of some concomitant drugs in the context of mBC and the resulting shortened life expectancy. Some treatments are undoubtedly necessary, such as for diabetes, hypothyroidism, or cardiovascular therapies. Studies have shown that diabetic patients appear to have poorer outcomes and higher mortality for all cancers [66, 67]. Likewise, cancer and hypertension are closely linked with shared pathophysiologic mechanisms and risk factors [68].

The indications for the use of other drugs, such as statins or proton-pump inhibitors (PPI), might be debatable [18]. Currently, there are no recommendations for the use of statins in patients with advanced cancer, and cardiovascular risk prediction models do not consider the shortened life expectancy [69, 70]. Physicians should therefore be aware of this issue and assess the need for statins on a patient-by-patient basis. Similarly, the long-term use of PPI can be problematic, as they may increase the risk of intestinal infections [71]. As patients with advanced cancer are often older and may be immunocompromised [72], drugs that may increase the risk of infection should be prescribed very carefully. In the present study, 26.9% of the patients took a PPI with only about half being on a concomitant non-steroidal anti-inflammatory drug (NSAID). However, without knowing the indication, it is difficult to argue whether there was overuse of PPI.

In our population of mBC patients, the largest drug group after mBC and supportive therapy were vitamins/minerals/supplements. In addition, a third of the patients reported to have ever used CAM in the course of their disease. Similarly, a systematic review described, that vitamins, herbs, and foods were the most commonly used types of CAM [73]. In the present study, vitamins and minerals comprised, among others, calcium and vitamin D3, which might be considered part of the mBC or supportive therapy. BC therapies such as aromatase inhibitors can cause loss of bone mineral density [74]. Therefore, clinical guidelines recommend the concomitant uptake of vitamin D3 and calcium [3].

Milic et al. (2015) investigated the patient-reported drug burden in mBC [18]. Similarly, they found a median number of six concomitant drugs per patient. However, other than in our analysis, they reported that the largest proportion of drugs comprised symptomatic/supportive therapy. Yet, they considered only oral anticancer agents, which might be the reason for this difference. Comparable proportions have been described for cardiovascular drugs, PPI, psychiatric drugs, and drugs for the endocrine system [18]. Cashman et al. (2010) analysed the treatment of comorbidities in patients (male and female) ≥ 65 years with different advanced cancers [16]. They found a median number of seven concomitant drugs, mostly including drugs for the cardiovascular system. This was also the largest drug group for comorbidities in our population. However, Cashman et al. (2010) did not include a sensitivity analysis for mBC patients [16].

We identified pMD in 52.7% of the patients. Several issues need to be addressed when interpreting these drugs. For the drugs from the STOPP/START Criteria, PRN medication must be considered. The absence of a PPI or laxative might be less problematic if the NSAID or opioid is not taken regularly, and therefore, the risk of potential side effects is reduced. Most pMD were identified according to the S3 Guidelines [3, 5]. However, not every observed pMD might actually be missing. Apart from antiemetics, most supportive therapies (e.g. antidiarrhoeals, therapy for neurotoxicity) are only prescribed if the patient experiences the respective side effects [3, 5]. Likewise, for aromatase inhibitors the S3 Guidelines recommends supplementation with vitamin D3 and calcium to reduce the risk for loss of bone mineral density and thus osteoporosis or fractures [3]. However, the calcium dosages should be mostly covered by food intake. Bisphosphonates or denosumab should only be used if the bone mineral density falls below a T-Score of -1.5 [3]. In the eight patients who were not taking vitamin D3, a bisphosphonate or denosumab, the T-Score might have been higher than this cut-off. Last, for all pMD, the documentation of the medication in the routine medical records might not have been complete. Particularly in mBC therapy, physicians should be aware of common potential side effects, such as nausea, emesis, diarrhoea, neurotoxicity or osteoporosis and take timely action.

Eventually, mBC patients might benefit from close monitoring of their medication by their treating physicians. Furthermore, they might benefit from being involved in this evaluation in the frame of shared decision-making, leading to better knowledge about their therapies and strengthening patient participation [75, 76].

### Potential factors influencing polypharmacy and potentially inappropriate medication

In the subgroup analyses, we identified several factors that might be associated with polypharmacy, PIM, pDDI, and pMD. These factors included, among others, older age, living alone, specific metastatic sites and treatment recommendations, and poor QoL. With regard to the reported side effects, patients who were at least slightly impaired by infections, side effects of the kidney or urinary tract, or side effects of the nervous system, took more concomitant drugs or were more prone to polypharmacy than patients who experienced other side effects. These factors are consistent with possible explanatory mechanisms described in current literature [9, 11–17, 77–86]. Considering these factors when evaluating the medication of mBC patients might help reduce their drug burden and the risk of potential adverse drug reactions.

### Strengths and limitations

To our knowledge, this was the first study to evaluate medication patterns and potential influencing factors in a German cohort of mBC patients using data from routine medical records. The major strength results from the reliability of the data source, as the information on the medication was directly extracted from routine medical records.

Our mBC cohort is comparable to the general population of BC patients in terms of HR and HER2 status expression (see Table 1) [87–89]. Furthermore, the patterns of metastatic sites (see Table 1) were comparable to those described in current literature [90, 91]. In addition, the observed CCI (see Table 1) was similar to observed values in other (m)BC cohorts [92, 93]. However, patients in our study cohort were younger than most mBC patients in literature [6, 7]. This might imply that the patients in our cohort might have more aggressive tumours [94]. In addition, we included patients with both, prevalent and newly diagnosed advanced or mBC. Thereby, the patients could be at any point in the course of therapy. This might have resulted in a heterogeneous cohort, as drug use at the start of the mBC therapy might differ from drug use at a later time point in therapy. However, this heterogeneity reflects the patient collective presenting in clinical routine.

A major limitation, resulting from the use of routine data, is that clinical data on indications were not available for non-mBC therapies. Therefore, implicit PIM criteria could not be applied to the present cohort [45]. Likewise, DDI detection tools or software [95] could not be applied. On the one hand, this may have led to an underestimation of PIM, pDDI, and pMD. On the other hand, an overestimation – for certain factors – is possible, since a patient’s specific situation may justify certain inappropriate prescriptions. Moreover, using explicit tools we were unable to assess the severity of pDDI. Hence, the use of explicit tools can only lead to assumptions of drug treatment quality [45].

In addition, these criteria only apply for patients ≥ 65 years [46–48]. However, it has been argued that the criteria can be applied to all age groups, since, morbidity and/or comorbidity is more important than age, in general [96]. Furthermore, patients may take additional OTC drugs that are not documented in the routine data. This might have also contributed to an underestimation of CAM, PIM, and pDDI. Hence, further studies should investigate in detail on the use of OTC drugs in mBC patients using more extensive medication lists.

Furthermore, due to the cross-sectional nature of our study, we were unable to determine whether patients were prescribed some drugs on a long or short term basis. Information on other than the mBC therapy were not continuously available for other time points in the routine medical records. In general, the absence of continuous medication plans in the patients’ medication history is a common problem in Germany.

Last, due to the small sample size, we could not identify trends or potential influencing factors for PIM or pDDI, nor could we statistically test the differences found in polypharmacy or pMD. Larger studies should examine patient- or disease-related factors, as PIM and pDDI may cause severe adverse events, reduce mBC patients’ quality of life, and adherence to drug therapy [16, 18].

### Conclusion

Despite the small size of our cohort, we found evidence for the relevance of polypharmacy, PIM, pDDI, and pMD in patients with mBC. More than a quarter of the documented drugs represent treatment for comorbidities or CAM. These drugs might not only be associated with additional risk of side effects, PIM use, pDDI, or pMD, yet might also contribute to low medication adherence, higher medication costs, and impaired QoL. Considering the severity and burden of mBC itself and the predicted life expectancy, mBC patients might benefit from close monitoring of their medication by their treating physicians. Larger studies are needed to evaluate the relationship between drug burden, PIM, pDDI, or pMD, and adherence to mBC therapy and QoL.

## Supplementary Information

Supplementary Material 1.

Supplementary Material 2.

Supplementary Material 3.

Supplementary Material 4.

## Data Availability

No datasets were generated or analysed during the current study.

## Abbreviations

BC: Breast cancer
BZD: Benzodiazepines
CAM: Complementary or alternative medicine
CCI: Charlson Comorbidity Index
CNS: Central nervous system
HER2: Human epidermal growth factor receptor 2
HR: Hormonal receptor
mBC: Metastatic breast cancer
NSAID: Non-steroidal anti-inflammatory drug
OTC: Over-the-counter
pDDI: Potential drug-drug interaction(s)
PIM: Potentially inappropriate medication(s)
pMD: Potentially missing drug(s)
PPI: Proton-pump inhibitor(s)
PRN: Pro-re-nata (= on-demand medication)
PROs: Patient reported outcomes
PROMIS-PF-4a: PROMIS® v2.0 Physical Function SF 4a
QoL: Quality of life
