# Supplementary material for: Medication patterns and potentially inappropriate medication in patients with metastatic breast cancer: results of the BRE-BY-MED study
Source: BMC Cancer. 2025 Jan 22;25:125. doi: 10.1186/s12885-025-13548-8 (PMC11756166; doi:10.1186/s12885-025-13548-8)
Supplement: Supplementary file 1 — Supplementary Material 1. [file 12885_2025_13548_MOESM1_ESM.docx]

*Supplement table. Patient questionnaire on impairment caused by side effects.*

| **How much do you feel impaired by the following (possible) side effects of your medication?**  ***(Please check only one box per side effect)*** |
| --- |

| 1. Infections and parasitic diseases (e.g. cystitis, sinusitis, blood poisoning, fungal infections) | | | | |
| --- | --- | --- | --- | --- |
| ***not at all*** | ***slight*** | ***moderate*** | ***considerable*** | ***severe*** |
|  |  |  |  |  |

| 1. Metabolic and nutritional disorders (e.g. weight loss, loss of appetite) | | | | |
| --- | --- | --- | --- | --- |
| ***not at all*** | ***slight*** | ***moderate*** | ***considerable*** | ***severe*** |
|  |  |  |  |  |

| 1. Side effects affecting the cardiovascular system (e.g. hot flushes with reddening of the skin, palpitations/fluttering of the heart, chest tightness, high blood pressure, swelling of the skin, phlebitis) | | | | |
| --- | --- | --- | --- | --- |
| ***not at all*** | ***slight*** | ***moderate*** | ***considerable*** | ***severe*** |
|  |  |  |  |  |

| 1. Side effects affecting the kidneys and/or urinary tract (e.g. difficult or increased urination, urinary incontinence) | | | | |
| --- | --- | --- | --- | --- |
| ***not at all*** | ***slight*** | ***moderate*** | ***considerable*** | ***severe*** |
|  |  |  |  |  |

| 1. Side effects that affect the respiratory tract (e.g. cough, asthma, shortness of breath) | | | | |
| --- | --- | --- | --- | --- |
| ***not at all*** | ***slight*** | ***moderate*** | ***considerable*** | ***severe*** |
|  |  |  |  |  |

| 1. Side effects that affect the eyes (e.g. dry eyes, conjunctivitis, increased lacrimation) | | | | |
| --- | --- | --- | --- | --- |
| ***not at all*** | ***slight*** | ***moderate*** | ***considerable*** | ***severe*** |
|  |  |  |  |  |

| 1. Side effects affecting the nervous system (e.g. tremors, dizziness, headaches, taste disturbance) | | | | |
| --- | --- | --- | --- | --- |
| ***not at all*** | ***slight*** | ***moderate*** | ***considerable*** | ***severe*** |
|  |  |  |  |  |

| 1. Side effects affecting the gastrointestinal tract (e.g. nausea, vomiting, diarrhoea, constipation) | | | | |
| --- | --- | --- | --- | --- |
| ***not at all*** | ***slight*** | ***moderate*** | ***considerable*** | ***severe*** |
|  |  |  |  |  |

| 1. Side effects that affect the skin (e.g. reddening of the skin, nail changes, hair loss) | | | | |
| --- | --- | --- | --- | --- |
| ***not at all*** | ***slight*** | ***moderate*** | ***considerable*** | ***severe*** |
|  |  |  |  |  |

| 1. Side effects affecting the muscles and/or bones (e.g. muscle pain, joint pain, pain in the extremities, bone pain, back pain) | | | | |
| --- | --- | --- | --- | --- |
| ***not at all*** | ***slight*** | ***moderate*** | ***considerable*** | ***severe*** |
|  |  |  |  |  |

| 1. Other side effects: __________________________________________________ | | | | |
| --- | --- | --- | --- | --- |
| ***not at all*** | ***slight*** | ***moderate*** | ***considerable*** | ***very*** |
|  |  |  |  |  |
