# Supplementary material for: Medication patterns and potentially inappropriate medication in patients with metastatic breast cancer: results of the BRE-BY-MED study
Source: BMC Cancer. 2025 Jan 22;25:125. doi: 10.1186/s12885-025-13548-8 (PMC11756166; doi:10.1186/s12885-025-13548-8)
Supplement: Supplementary file 2 — Supplementary Material 2. [file 12885_2025_13548_MOESM2_ESM.docx]

*Supplement table 2. List of applied explicit potential drug-drug interactions.*

| **Drug 1** | | **Drug 2** | | **Reason** | **Originating list** | |
| --- | --- | --- | --- | --- | --- | --- |
| **Drug / drug type** | **ATC code** | **Drug / drug type** | **ATC code** |  | **STOPP/START Criteria** | **Beers Criteria** |
| Non-steroidal anti-inflammatory drug (NSAID) | M01A | Vitamin K Antagonist | B01AA | Risk of major GI bleeding | x |  |
|  | M01A | Factor Xa Inhibitors | B01AF | Risk of major GI bleeding | x |  |
|  | M01A | Direct Thrombin Inhibitor | B01AE | Risk of major GI bleeding | x |  |
|  | M01A | ACE-Inhibitor and diuretic | C09A and C03 | "Triple Whammy" | x |  |
| Metoclopramide | A03FA01 | Anti-Parkinson drugs | N04 | Risk of exacerbating Parkinsonian symptoms | x |  |
| Beta blocker | C07A | Cardio-selective calcium channel blocker (Verapamil, Diltiazem) | C08D | Risk of atrioventricular block, myocardial depression | x |  |
| Direct thrombin inhibitor | B01AE | Cardio-selective calcium channel blocker (verapamil, diltiazem) |  | Risk of bleeding | x |  |
| Apixaban, dabigatran, edoxaban, rivaroxaban | B01AE07, B01AF01, B01AF02, B01AF03 | P-glycoprotein drug efflux pump inhibitors (e.g., amiodarone, azithromycin, carvedilol, cyclosporin, dronedarone, itraconazole, ketoconazole (systemic), macrolides, quinine, ranolazine, tamoxifen, ticagrelor, verapamil) | C01BD01, J01FA10, C07AG02, L04AD01, C01BD07, J02AC02, J02AB02, J01FA, C05AF01, M09AA02, P01BC01, C01EB18, B01AC24, C08DA01, L02BA01 | Risk of bleeding | x |  |
| Opioid | N02A | Benzodiazepines | N05BA | Increased risk of overdose and adverse events |  | x |
|  |  | Gabapentin, pregabaline | N02BF | Increased risk of overdose and adverse events |  | x |
| ≥ 2 more anticholinergic drugs | N04A, G04B | / | / | Increased risk of cognitive decline, delirium, and falls or fractures |  | x |
| ≥ 2 more RAS inhibitors | C09 | / | / | Increased risk of hyperkalaemia |  | x |
| ≥ 3 CNS-active drugs (Antiepileptics, Gabapentinoids, Antidepressants (TCAs, SSRIs, and SNRIs), Antipsychotics, Benzodiazepines, Z-drugs, Opioids, Skeletal muscle relaxants)^$^ | N02A, N02BF, N03A, N05A, N05BA, N05CF, N06AA09, N06AB, N06AX16, N06AX21, M03B | / | / | Increased risk of falls and of fracture |  | x |
| *^$^ CNS = central nervous system, TCAs = tricyclic antidepressants , SSRIs = selective serotonin reuptake inhibitors, SNRIs = serotonin–norepinephrine reuptake inhibitors* | | | | | | |
