# Supplementary material for: Medication patterns and potentially inappropriate medication in patients with metastatic breast cancer: results of the BRE-BY-MED study
Source: BMC Cancer. 2025 Jan 22;25:125. doi: 10.1186/s12885-025-13548-8 (PMC11756166; doi:10.1186/s12885-025-13548-8)
Supplement: Supplementary file 3 — Supplementary Material 3. [file 12885_2025_13548_MOESM3_ESM.docx]

*Supplement table 3. List of applied explicit potential missing drugs.*

| **Present drug/ drug type** | | **Missing drug** | | **Originating list** | |
| --- | --- | --- | --- | --- | --- |
| **Drug/ drug type** | **ATC code** | **Drug/ drug type** | **ATC code** | **STOPP-START Criteria** | **S3 Guidelines BC and S3 Guideline supportive therapy in cancer** |
| Non-steroidal anti-inflammatory drug (NSAID) | M01A | PPI | A02BC | x |  |
| Opioid | N02A | Laxative | A06A | x |  |
| Corticosteroid | H02A | Bisphosphonate and vitamin D3 | M05BA and A11CC | x |  |
| Osteoporosis medication | H05BA/ M05BX04/ H05AA02/ M05BX03/ M05BX53 | Vitamin D3 and calcium | A11CC and A12AA | x |  |
| Methotrexate | L04AX03, L01BA01 | Folic acid supplement | B03BB01 | x |  |
| Cyclophosphamid | L01AA01 | Antiemetic drug (ondansetron/ dexamethasone/ metoclopramide/ dimenhydrinate/ olanzapine/ haloperidol/ levomepromazine/ lorazepam/ alprazolam) | A04AA01/ H02AB02/ A03FA01/ A04AB02/ N05AH03/ N05AD01/ N05AA02/ N05BA06/ N05BA12 |  | x |
| Carboplatin | L01XA02 |  |  |  | x |
| Doxorubicin | L01DB01 |  |  |  | x |
| Epirubicin | L01DB03 |  |  |  | x |
| Capecitabine | L01BC06 |  |  |  | x |
| Docetaxel | L01CD02 |  |  |  | x |
| Eribulin | L01XX41 |  |  |  | x |
| Paclitaxel/nab-Paclitaxel | L01CD01 |  |  |  | x |
| Pertuzumab | L01FD02 |  |  |  | x |
| Lapatinib | L01EH01 |  |  |  | x |
| Trastuzumab-Emtansin | L01FD03 |  |  |  | x |
| Paclitaxel | L01CD01 | Antineurotoxic drug (venlafaxine/ amitriptyline/ gabapentin/ pregabaline) | N06AX16/ N06AA09/ N02BF01/ N02BF02 |  | x |
| Docetaxel | L01CD02 |  |  |  | x |
| Docetaxel | L01CD02 | Loperamide (antidiarroic agent) | A07DA03 |  | x |
| Bevacizumab | L01FG01 |  |  |  | x |
| Lapatinib | L01EH01 |  |  |  | x |
| Aromatase inhibitor | L02BG | Calcium | A12AA |  | x |
|  |  | Vitamin D3 | A11CC05 |  | x |
|  |  | Bisphosphonate/ denosumab | M05BA, M05BX04 |  | x |
