# Supplementary material for: Medication patterns and potentially inappropriate medication in patients with metastatic breast cancer: results of the BRE-BY-MED study
Source: BMC Cancer. 2025 Jan 22;25:125. doi: 10.1186/s12885-025-13548-8 (PMC11756166; doi:10.1186/s12885-025-13548-8)
Supplement: Supplementary file 4 — Supplementary Material 4. [file 12885_2025_13548_MOESM4_ESM.docx]

**Supplementary material 4**

**Medication patterns and drug types – detailed description of the observed drug types**

Therapy for metastatic breast cancer (mBC) comprised antineoplastic agents (n=127, 19.0%) (i.e. alkylating agents, pyrimidine analogues, taxanes, anthracyclines, human epidermal growth factor receptor 2 (HER2) inhibitors, monoclonal antibodies, platinum-based drugs, PARP inhibitors, eribulin), endocrine therapy (n=113, 17.0%) (i.e. aromatase inhibitors, CDK4/6 inhibitors, GnRH analogues, antiestrogens), drugs affecting bone structure and mineralisation (n=51, 7.6%) (i.e. bisphosphonates, denosumab), and immunostimulants (n=3, 0.4%) (i.e. colony-stimulating factors).

Symptomatic/supportive therapy comprised analgesics (n=69, 10.3%) (i.e. opioids, metamizole, paracetamole, gabapentinoids, migraine medication), antiemetics (n=44, 6.6%) (i.e. metoclopramide, ondansetron, dimenhydrinate, dexamethasone, methylprednisolone), laxatives (n=5, 0.7%) (i.e. macrogol, bisacodyl), antidiarrhoeals (n=2, 0.3%) (i.e. loperamide). Supportive therapy for neurotoxicity (according to the German S3 Guideline for Diagnostic, Treatment and Follow-up Care of Breast Cancer [Leitlinienprogramm Onkologie (Deutsche Krebsgesellschaft, Deutsche Krebshilfe, AWMF); Version 4.3; 2020], i.e. venlafaxine, amitriptyline, gabapentin, pregabaline) were either categorised as analgesics or psychoanaleptics. Psychiatric drugs comprised psychoanaleptics (n=11, 1.7%) (i.e. antidepressants) and psycholeptics (n=10, 1.5%) (i.e. antipsychotics, anxiolytics, zolpidem). Drugs for the CNS system solely comprised antiepileptics (n=3, 0.4%).

Vitamins/minerals/supplements comprised minerals (n=45, 6.7%) (i.e. calcium, magnesium, sodium, potassium), vitamins (n=29, 4.2%) (i.e. vitamin D3) and other nutritional supplements (n=7, 1.1%).

Drugs for the cardiovascular system comprised antihypertensive drugs (n=30, 4.5%) (i.e. antiadrenergic agents, diuretics, agents acting on the renin-angiotensin system), beta-blocking agents (n=12, 1.8%) and calcium channel blockers (n=3, 0.4%), antithrombotic agents (n=18, 2.7%) (i.e. acetylsalicylic acid, enoxaparin), lipid modifying agents (n=7, 1.1%) (i.e. statins, ezetimibe), antianaemic agents (n=5, 0.7%) (i.e. folic acid, iron supplements), and rutoside, a vasoprotective agent.

Drugs for the gastrointestinal system comprised proton pump inhibitors (n=25, 3.7%), anti-diabetics (n=2, 0.3%), and a substance for bile and liver therapy. Drugs for the endocrine system solely comprised drugs for thyroid therapy (n=26, 4.0%) (i.e. levothyroxine, iodine). Drugs for the respiratory system solely comprised drugs for obstructive airway diseases (n=11, 1.7%) (i.e. inhalants).

Other drugs comprised homeopathic agents (n=3, 0.4%), gynaecologic agents (n=3, 0.4%) (i.e. an anti-infective, a substance for urinary frequency and incontinence, black cohosh (*Cimicifuga racemosa [L.] Nutt.*)), an antimycotic agent, and an antigout agent.
